# Increased Uterine NK cell numbers and perforin expression during the implantation phase in IVF Cycles with GnRH Antagonist Protocol

**DOI:** 10.1038/srep39912

**Published:** 2017-01-03

**Authors:** Bufang Xu, Jingwen Wang, Lan Xia, Dan Zhang, Xian Wu, Aijun Zhang

**Affiliations:** 1 Reproductive Medical Center of Ruijin Hospital, School of Medicine, Shanghai Jiao Tong University, Shanghai 200025, China; 2 Shanghai Key Laboratory of Reproductive Medicine, School of Medicine, Shanghai Jiao Tong University, Shanghai 200025, China.

## Abstract

GnRH antagonist negatively affects endometrial receptivity in *in vitro* fertilization (IVF) cycles, however, its underlying mechanism remains unclear. To explore its target molecules, we studied endometria in the window phase of fixed GnRH antagonist, low-dose flexible GnRH antagonist, GnRH agonist long protocol, and untreated control groups. There were 384 differentially expressed genes (DEGs) in the fixed antagonist group with greater than twofold expression change compared with the control group and 197 DEGs between the fixed antagonist and agonist groups, the majority of which were associated with the natural killer (NK) cell-mediated cytotoxicity pathway. We then analysed the PRF1 and FASLG protein levels. The perforin level were significantly higher in both the antagonist groups than in other two groups, and was higher in the fixed antagonist group. Similarly, the uNK cell numbers were higher in the antagonist groups, and the highest uNK cell number occurred in the fixed group (p < 0.05). No significant differences existed in the Fas ligand levels and apoptosis rates among the three treatment groups, but were higher in the treatment groups than the control group. Together, these data indicate that GnRH antagonist may increase the uNK cell numbers and perforin expression, and this effect may be dose-dependent.

Gonadotropin-releasing hormone (GnRH) antagonists were first introduced into *in vitro* fertilization-embryo transfer (IVF-ET) protocols in 2001 and have increased in popularity in recent years because they have several advantages when used in IVF-ET, including a lack of hypo-oestrogenism, short treatment duration, lower gonadotropin requirement, and potential reduction in the incidence of severe ovarian hyperstimulation syndrome^1^,^2^. However, in many studies, GnRH antagonist-stimulated cycles are associated with lower implantation, clinical pregnancy, and ongoing pregnancy/live birth rates than GnRH-agonist long protocol cycles when a standardized number of embryos are transferred^3^,^4^,^5^.

Embryo implantation is a highly coordinated event influenced by embryo quality and endometrial receptivity. In the past few years, studies have investigated the impact of GnRH antagonist on clinical pregnancy. Some studies have reported no significant differences in clinical outcomes in frozen-thawed embryo transfer cycles between patients treated with GnRH agonist and GnRH antagonist^6^,^7^. Thus far, no significant differences have been found between the two protocols with respect to the levels of specific biomarkers in follicular fluid, oocyte dysmorphisms, oocyte quality, and embryo quality^8^,^9^,^10^. This indicates that endometrial receptivity, but not embryo quality, may be a factor contributing to the low pregnancy rates observed in patients treated with GnRH antagonist^11^. Recent findings have shown that the luteal phase single-dose GnRH agonist protocol can improve clinical outcomes after *in vitro* fertilization/intracytoplasmic sperm injection (IVF/ICSI), suggesting that the GnRH agonist may exert beneficial effects on the endometrium as endometrial epithelial and stromal cells express GnRH receptors (GnRHRs)^12^,^13^. Conversely, GnRH antagonists may negatively influence the endometrium via GnRHR^14^,^15^.

Thus far, several approaches such as histological evaluation^16^, gene expression analysis^17^, and endometrial transcriptome study^18^ have been investigated for the effect of GnRH antagonist on the endometrium during the implantation window. Moreover, biochemical factors of presumed importance in endometrial receptivity, such as HOXA10, were significantly decreased in endometrial stromal cells^15^. However, the key factors involved in this process remain unclear. According to our research, we explored the factors by which GnRH antagonist affects the endometrial receptivity, so as to provide theoretical support for the underlying mechanisms of this phenomenon and to optimize its application in ovarian stimulation in IVF in the future.

## Results

### Demographic characteristics

The demographic characteristics of the women did not significantly differ among the four groups, and the total dosages and duration of Cetrotide used were significantly higher in the fixed antagonist group than in the flexible antagonist group (P < 0.01, Table 1).

### Microarray expression profiling

**Figure 1:**
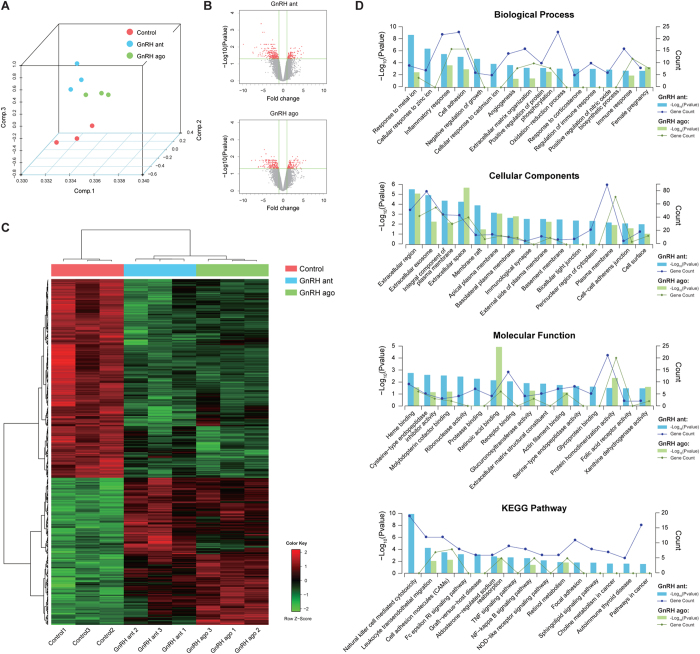
Feature Extraction from microarray data. (**A**) 3D scatter plot of Principal Component Analysis (PCA) results. Three samples in the control group (red colour), three samples in the GnRH antagonist group (blue colour), and three samples in the GnRH agonist group (green colour) were divided into three significant clusters. (**B**) Volcano plot of all expressed genes. Compared with control group, 384 differentially expressed genes (DEGs) in the antagonist group and 363 DEGs in the agonist group (red colour) with > twofold change and an adjusted *P* value of <0.05. (**C**) Hierarchical clustering of DEGs. A total of 384 DEGs in antagonist group and 363 DEGs in agonist group were indicated. (**D**) Functional enrichment results of DEGs, including KEGG pathways and GO. *P* values and gene count of each term are shown as bar plots and lines, respectively.

According to the normalized data, principal component analysis (PCA) showed three significant clusters for the fixed GnRH antagonist, long GnRH agonist, and untreated natural (control) groups (Fig. 1A). Among all the probe sets, in the antagonist group, 384 differentially expressed genes (DEGs) exhibited a fold change of ≥2 and an adjusted P value of ≤0.05. Of these, 144 were upregulated and 240 were down regulated. Similarly, 363 DEGs were identified in the long agonist group. There were 197 DEGs that had changed in the antagonist group but not in the agonist group. (Fig. 1B). All DEGs were clustered using a hierarchical clustering algorithm (Fig. 1C). The result showed that the gene expression pattern of the antagonist group significantly differed from that of the control and agonist groups. Using Gene Ontology (GO) analysis, these genes were clustered into groups on the basis of their biological processes, cellular components, and molecular functions. A large proportion of the genes were involved in cell adhesion. Other groups related to implantation included those involved in responses to wounding, corticosteroid stimulus, and female pregnancy. In addition, Kyoto Encyclopedia of Genes and Genomes (KEGG) pathway analysis showed that the majority of these DEGs were associated with the NK cell-mediated cytotoxicity pathway in the antagonist group but only few of the DEGs in the agonist group were associated with this pathway. Other groups included genes involved in leukocyte transendothelial migration, cell adhesion molecules (CAMs), and apoptosis (Fig. 1D).

### Validation of profiling data using quantitative reverse transcriptase-polymerase chain reaction (qRT–PCR)

**Figure 2:**
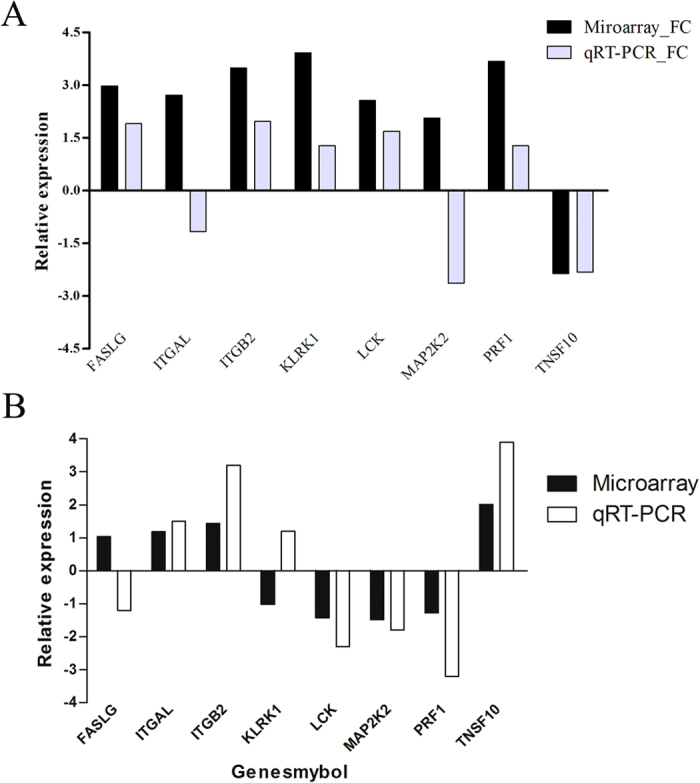
Relative expression of microarray and qRT-PCR. (**A**) The majority of genes in the NK cell-mediated cytotoxicity pathway in antagonist group compared with the control group. (**B**) The majority of genes in the NK cell-mediated cytotoxicity pathway in the agonist group compared with the antagonist groups.

There were 21 differentially expressed genes (FC > 2) in the NK cell-mediated cytotoxicity pathway in GnRH antagonist group compared with the natural group. Eight genes (*PRF1, FASLG, TNSF10, ITGAL, ITGB2, KLRK1, LCK and MAP2K2*), playing an important role in embryo implantation, were chosen to be validated by qRT-PCR. The results showed that, *TNSF10* was the only one down regulated gene while other genes were all up regulated. The qRT-PCR results were consistent with the cDNA microarray findings (Fig. 2A). On the other hand, in the agonist group, the *FASLG* level was 1.04-fold upregulated in microarray and 1.2-fold downregulated in qRT-PCR, while the *PRF1* mRNA level was 1.27-fold downregulated in microarray and 3.2-fold downregulated in qRT-PCR (Fig. 2B). Levels of *FASLG* and *PRF1* in the antagonist groups were higher than those in the agonist group, respectively. *PRF1* and *FASLG* were the two upstream genes in the NK cell-mediated cytotoxicity pathway, therefore, we further examined the protein expression of the two genes.

### Expression of FasL, perforin, CD56^bright^, GnRHR, and apoptosis rate

**Figure 3:**
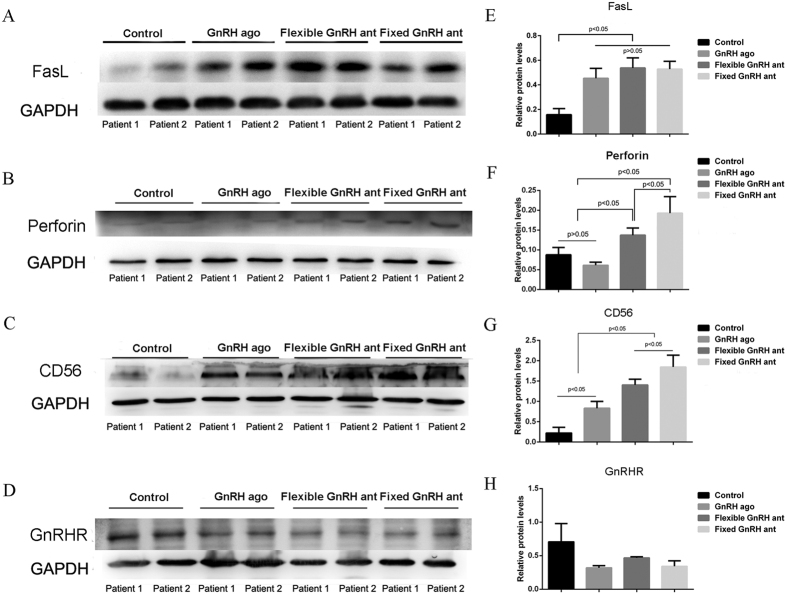
Representative images and quantification of FasL (**A**,**E**), perforin (**B**,**F**), uNK cells (**C**,**G**), and GnRHR (**D**,**H**) of four groups assayed by Western blot. All data are mean values ± standard deviation, and considered statistically significant at P < 0.05. Cropped gels and blots are displayed, and full-length gels and blots are included in the Supplementary Fig. S1.

**Figure 4:**
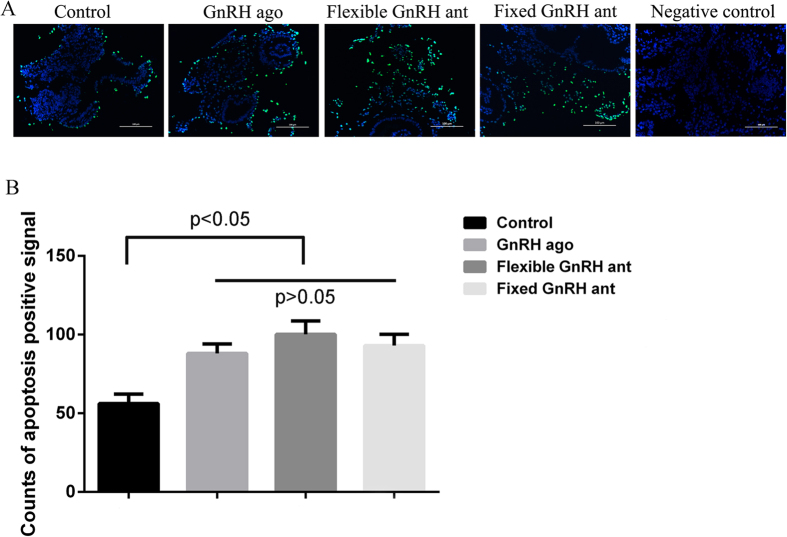
Representative images of apoptotic cells in the four groups. (**A**) Cells were examined by the TUNEL assay to detect apoptosis (green), and counter stained with DAPI to detect nuclei (blue). (**B**) Apoptosis rates in treatment groups were higher than that in the control group, but the differences between the treatment groups were not statistically significant. The number of TUNEL-positive cells were counted in 3 fields for each slide and analyzed. All data are mean values ± standard deviation, and considered statistically significant at P < 0.05.

**Figure 5:**
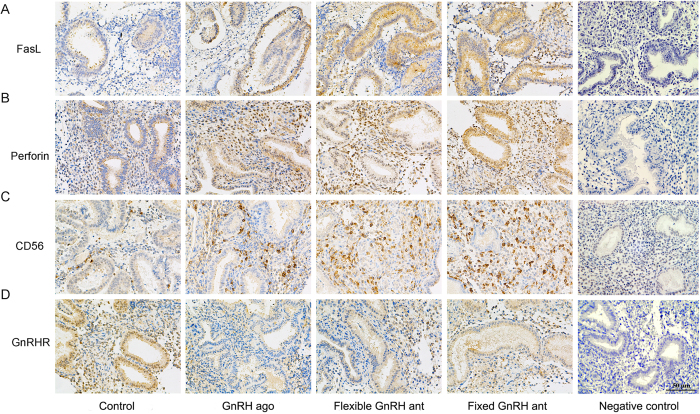
Representative images of FasL (**A**), perforin (**B**), uNK cells (**C**), and GnRHR (**D**) of four groups using immunohistochemical assay.

Results of the Western blot analysis showed that the Fas Ligand (FasL) expression levels in the three treatment groups (fixed GnRH antagonist group, flexible GnRH antagonist group and GnRH agonist long group) were higher than those in the control group, but the differences in the FasL expression level between the treatment groups were not statistically significant (Fig. 3, see Supplementary Fig. S1). Similar results were found with regard to the apoptosis rates (Fig. 4). The expression levels of perforin and CD56^bright^, the phenotypic markers of uNK cells, were significantly higher in the two antagonist treatment groups than in the agonist treatment and control groups, and it was notably higher in the fixed treatment group than in the flexible treatment group (P < 0.05 in both cases). The exact gray values of CD56^bright^ in four groups were as follows: fixed GnRH antagonist group (1.84 ± 0.29), flexible GnRH antagonist group (1.41 ± 0.14), GnRH agonist group (0.83 ± 0.17), and natural group (0.22 ± 0.14). In addition, the results revealed no differences in the GnRHR expression levels among the four groups. The results of the immunohistochemical analysis were roughly consistent with those of the Western blot analysis (Figs 3 and 5).

## Discussion

In recent years, the GnRH antagonist protocol has significantly improved the flexibility and security of clinical applications compared with the GnRH agonist long protocol, and this antagonist protocol has been widely used in IVF cycles. Despite this, the antagonist protocol is associated with lower clinical pregnancy rate. Embryo quality as well as endometrial receptivity are well known to be key factors influencing embryo implantation. Many researchers have demonstrated that GnRH antagonist negatively affected endometrial receptivity but not embryo quality; however, the mechanism by which the GnRH antagonist influences endometrial receptivity remains to be understood. In this study, we found that the numbers of uNK cells and the perforin expression level were increased during the implantation phase in the patients undergoing the GnRH antagonist protocol.

We carried out gene expression analysis to identify the factors influenced by GnRH antagonist. A total of 384 genes exhibited a difference in expression of more than 2× during the window phase in the fixed GnRH antagonist protocols compared with the control cycles, while the expression levels of 197 genes differed between the antagonist and agonist long protocols. KEGG pathway analysis showed that the NK cell-mediated cytotoxicity pathway was highlighted in the antagonist groups than in the agonist group. We further detected the expression of the main genes in this pathway using qRT-PCR, and found that the expression of most genes (*FASLG, ITGB2, KLRK1, LCK*, and *PRF1*) were up-regulated.

Human uNK cells, which are identified by the CD56^bright^ phenotype, are the most important lymphocytes in the uterus. As a key component of innate immunity, they not only participate in the early phase of immune responses against certain viruses, parasites, and microbial pathogens^19^, but also play an important role in the process of human pregnancy^20^. Substantial amounts of data support that uNK cells help to regulate pregnancy maintenance via several mechanisms, including the production of a variety of cytokines capable of directly influencing trophoblast growth and hormone production as well as implantation and vascularization of the decidua, producing immune modulatory proteins involved in immune regulation at the maternal-foetal interface, and control of trophoblast invasion via cell-mediated cytotoxicity^21^,^22^. The number of uNK cells changes during the normal menstrual cycle and human pregnancy. For example, it notably increases during the secretory phase, and reaches a peak during early pregnancy^23^. The expanded uNK cell numbers during pregnancy may play an important role in embryo implantation as well as in the creation of local immunosuppression at the maternal-foetal interface^24^. However, both infertile women and women with recurrent spontaneous abortions have been reported to have significantly abnormal elevation of NK levels^25^. Several researchers^26^,^27^,^28^,^29^,^30^ have shown that the disturbance of uNK cells during the receptive phase could lead to implantation failure. Our results also showed that the number of uNK cells during the implantation phase was significantly higher in the fixed antagonist group than that in the flexible antagonist group, and the numbers of uNK cells in both these groups were notably higher than those in the agonist and control groups. As unsatisfactory clinical outcomes have been previously observed in GnRH antagonist-stimulated cycles^3^,^4^,^5^, these results suggest that inadequate control of uNK cell proliferation might be harmful for embryo implantation.

On the other hand, many studies have shown that during the early stages of normal pregnancy, sex hormones such as oestrogen, progesterone, and prolactin may enhance the migration of peripheral CD56^bright^ NK cells to the uterus from a subset of peripheral blood NK cells. In this study, the oestrogen levels were significantly higher in the three treatment groups than in the control group, suggesting that higher numbers of uNK cells might be associated with higher oestrogen levels. In addition, while there were no significant difference in the oestrogen levels among the three treatment groups, the number of uNK cells was notably higher in the antagonist groups than in the agonist group. Moreover, the number of uNK cells was higher in the fixed antagonist group than in the flexible antagonist group, indicating that apart from oestrogen, GnRH antagonist may enhance the number of uNK cells in a dose-dependent manner, which is in accordance with previous reports that GnRH antagonists have a dose-dependent effect on implantation^31^,^32^. Taken together, under the influence of GnRH antagonist, the dramatic increase of the uNK cells population during the secretory phase may be one of the reasons that the pregnancy rates in patients in the GnRH antagonist groups were lower than that in patients in the GnRH agonist group.

uNK cells are well known to promote cell killing via two different signal transduction pathways. One is the granule exocytosis pathway mediated by cytotoxic granules such as perforin and granulysin, and the other is a death receptor pathway mediated by the ligation of cell death receptors on target cells, such as Fas and FasL. Therefore, we further determined the protein expression levels of *PRF1* and *FASLG*, two upstream genes in the NK cell-mediated cytotoxicity pathway.

Perforin is a cytotoxicity mediator released by cytoplasmic granules and is a major effector of NK cells. NK cell-mediated cytolysis is highly dependent on perforin, which induces lysis and promotes the death of target cells^33^. It has been reported that more than 95% of all decidual CD56^bright^ cells expressed perforin^34^,^35^, and appropriate perforin levels plays an important role in embryo implantation and maintaining normal human pregnancy. One study illustrated a slightly higher distribution of perforin-positive uNK cells in human sporadic miscarriages with a normal foetal chromosomal karyotype^36^, implying that higher perforin level might be harmful to the pregnancy. In this study, we found that the perforin level was significantly higher in the fixed antagonist group than in the flexible antagonist group, and the levels in both these groups were prominently higher than those in the agonist and control groups, with statistical significance. This finding suggested that GnRH antagonist might increase the perforin expression in IVF cycles in a dose-dependent manner. As we know, perforin is one of cytokines secreted by uNK cells, therefore, the main reason behind the phenomenon may be due to the increase of the uNK cell population. it may be also owing to the increased secrete function of uNK cells, which will be studied in the future.

FasL, another central regulator in the immune system, has been implicated in the maintenance of immune privileged sites. In normal pregnancy, it involves immune tolerance at the maternal–foetal interface, regulates trophoblast invasion during embryo implantation, and mediates apoptosis in susceptible cells via stimulation of caspase activity^24^. In addition, it has been reported that FasL-mediated apoptosis takes part in dynamic functional and structural changes in the bovine endometrium^37^, and that the absence of this factor at the maternal-foetal interface resulted in a marked reduction in fertility in mice^38^. The results suggest that FasL-mediated apoptosis is involved in the first trimester of pregnancy. In this study, the expression of FASLG was lower (–1.04×) by microarray and higher (1.2×) by qRT-PCR in the fixed GnRH antagonist group compared to the GnRH agonist group. However, the FasL expression level and the endometrial cell apoptosis rates were not observably different among the three treatment groups. These findings together indicate that the antagonist has little influence on the FasL expression or on FasL-mediated apoptosis. In addition, we also found that the FasL expression levels were markedly higher in the three treatment groups than in the control group, which might be attributable to the high level of E_2_. It has been reported that E_2_ enhanced the cytotoxic activity of YT-N17 (a human NK-like cell line) *in vitro* in a concentration-dependent manner^39^,^40^.

In conclusion, we demonstrated that the GnRH antagonist protocol may increase the uNK cell numbers and perforin expression, and this effect may be dose-dependent, which may be one of the reasons for the unsatisfactory clinical outcomes in the GnRH antagonist protocol in IVF. Therefore, appropriately reducing the GnRH antagonist dosage may help reduce its negative impact on endometrial receptivity. Further studies are required to investigate the mechanisms responsible for the roles of GnRH antagonist and perforin in endometrial receptivity in IVF cycles.

## Materials and Methods

### Patients

All the participants signed informed consents which were approved by the Shanghai Jiaotong University Committee on the Use of Human Subjects in Medical Research. Ethical approval was obtained from the Institutional Ethics Committee of the Ruijin Hospital, School of Medicine, Shanghai Jiao Tong University for all tissue collections and all experiments were performed in accordance with the guidelines and regulations of Practice Committee of American Society for Reproductive Medicine (ASRM). A total of 80 women, who were undergone IVF/ICSI treatment in Reproductive Medical Center of Ruijin Hospital, School of Medicine, Shanghai Jiao Tong University during January 2012 and December 2014, and gave up fresh embryo-transfer and received all-embryo cryopreservation due to personal reasons, for instance, on business, feeling uncomfortable on the embryo-transfer day, were enrolled into the study. Patients with endometriosis or polycystic ovary syndrome were excluded, and the following inclusion criteria were applied: 25 to 35 years of age, healthy, regular ovulatory cycles every 27–32 days, normal endocrine profile, normal serum levels of follicle stimulating hormone (FSH < 10 mIU/mL), luteinizing hormone (LH < 10 mIU/mL), and oestradiol (E2 < 50 pg/mL) on day 3 of the menstrual cycle, absence of abnormal ovarian or/and endometrial ultrasonographic features, and no use of contraceptive drugs or intrauterine devices within the last six months.

### Stimulation protocol

The patients were divided into four groups (n = 20 per group): natural cycles without treatment, GnRH agonist long protocol, flexible low-dose GnRH antagonist protocol, and fixed multi-dose GnRH antagonist protocol. Women with flexible low-dose GnRH antagonist protocol or fixed multi-dose GnRH antagonist protocol act as experimental group, and with natural cycle protocol or GnRH agonist protocol as control groups.

#### GnRH antagonist protocol

rFSH (Gonal-F, Merck Serono S.A., Switzerland) stimulation was initiated on day 2 of the menstrual cycle. The starting gonadotropin dose was determined according to the age, antral follicle count, basal FSH and E_2_ levels, and body mass index. This dose was adjusted after day 5 of stimulation, depending on the ovarian response evidenced by the E_2_ levels and ultrasound analysis.

#### Fixed protocol

Patients received 0.25 mg cetrorelix acetate/day (Cetrotide, Merck Serono SA, Switzerland) when the leading follicle reached 14 mm and continued daily until oocyte maturation.

#### Flexible protocol

Patients received 0.125 mg Cetrotide daily for serum LH levels of up to 7 IU/L or 0.25 mg Cetrotide daily for serum LH levels of up to 9 IU/L. Cetrotide administration continued daily until oocyte maturation. The total doses and duration of GnRH antagonist in flexible protocol were significantly lower than those in fixed protocol (Table 1), therefore the group was added to investigate the dose effect of GnRH antagonist on endometrial receptivity.

#### GnRH agonist long protocol

Patients received 0.1 mg triptorelin/S.C (Decapeptyl, Ipsen, Signes, France) daily for 2 weeks before anticipated menstruation. After pituitary downregulation was confirmed (indicated by serum E_2_ levels <50 pg/mL and endometrial thickness <5 mm), the patients were administered Gonal-F with 0.05 mg/S.C triptorelin daily until oocyte maturation was triggered. For all the three protocols, if three follicles reached a mean diameter of 17 mm, 5000 IU of hCG (Lizhu, Zhuhai, China) was administered intramuscularly. Oocyte retrieval was performed 35–36 h after human chorionic gonadotropin (hCG) injection by transvaginal ultrasound-guided single-lumen needle aspiration. Intracytoplasmic sperm injection (ICSI) was performed only in severe male factor infertility or previous fertilization failure. Luteal phase support with 600 mg of micronized progesterone (Crinone, Merck Serono S.A., Switzerland) was initiated on day 1 after oocyte retrieval till the biopsy day.

### Endometrial biopsy

Specimens were obtained via pipe suction curettage (Wallace) from the 20 women in untreated cycles on day LH + 7^41^ and from the other 60 women on day 7 after oocyte recovery. Tissue samples were washed thoroughly with sterile normal saline to remove excess blood and mucous. Some samples were flash frozen in liquid nitrogen for microarray, qRT-PCR, and Western blot, while other samples were fixed in 10% formalin for immunohistochemistry.

### Microarray studies

Agilent human gene expression 8*60 K (Design ID:039494) arrays were used for whole genome expression profiling of three groups: the control, GnRH agonist long protocol, and fixed GnRH antagonist groups, with three samples per group (Table 2). Total RNA was isolated using an RNeasy Mini Kit (Qiagen, Hilden, Germany) according to the manufacturer’s protocol. The RNA yield was measured with a NanoDrop ND-2000 (Thermo Fisher Scientific, Waltham, MA), and integrity assessed using Agilent Bioanalyzer 2100 (Agilent Technologies). Sample labelling, microarray hybridization, and washing were performed according to the manufacturer’s protocols. Briefly, total RNA was transcribed into double-stranded cDNA, synthesized into cRNA, and labelled with cyanine-3-CTP. Labelled cRNAs were hybridized onto the microarray. After washing, the arrays were scanned with the Agilent Scanner G2505C (Agilent Technologies).

Feature Extraction software (v10.7.1.1, Agilent) was applied to analyse array images and generate raw data for probe intensity. The raw data were normalised and background was adjusted using the quantile algorithm within the R package marray. Normalized data were analysed with a linear model for microarray data (LIMMA), and a modified *t* test incorporates the Benjamini-Hochberg multiple hypotheses correction technique^42^. DEGs were identified based on fold changes and adjusted P values. A fold change threshold of ≥2.0 and adjusted P value ≤ 0.05 were set for up- and downregulated genes. Functional enrichment analyses including GO and KEGG pathway enrichment were assessed using the DAVID database^43^ (http://david.abcc.ncifcrf.gov/). For functional enrichment analysis of DEGs, the background was set to the total list of genes expressed in humans. The statistically significant threshold level for enrichment analyses was P ≤ 0.05 (Benjamini and Hochberg corrected for multiple comparisons). Finally, PCA was carried out on the basis of all probe sets to investigate the difference in the gene expression profiles among the three groups. Hierarchical clustering was also performed to determine the expression patterns of DEGs using R package gplots. R 3.1.3 was used for data manipulation and visualization.

### mRNA analysis by quantitative RT–PCR

Total RNA was extracted from endometria using Trizol (Invitrogen), and the RNA was reverse transcribed with a cDNA Reverse Transcription Kit (TOYOBO, Osaka, Japan). Real-time quantitative PCR was performed using an TAKARA Analyzer with SYBR Green Master Mix (Takara, Shiga, Japan) with primers as Table 3. The mRNA expression levels were normalized to the expression level of *GAPDH* and calculated using the 2^−ΔΔ^Ct method^44^.

### Western blot analysis

Endometrial samples were ground in a homogenizers with RIPA lysis buffer and protease inhibitor cocktail (Thermo Fisher Scientific, USA) on ice. Protein extracts were obtained by centrifuging the samples at 12,000× *g* for 10 min at 4 °C. The protein concentrations were determined using a BCA Protein Assay Kit (Beyotime, China). The protein samples (80 μg per well) were then separated by 12% sodium dodecyl sulphate-polyacrylamide gel electrophoresis (SDS-PAGE), and the resolved proteins were then transferred onto polyvinylidene difluoride (PVDF) membranes (Millipore, Billerica, MA, USA), and blocked with 5% nonfat milk in Tris-buffered saline (TBS) for 1 h. For immunoblotting, proteins were incubated overnight at 4 °C with primary antibodies against perforin (Santa Cruz), FasL (R&D), GnRHR (Abcam), and CD56 (R&D), and incubated with corresponding secondary horseradish peroxidase (HRP)-conjugated antibodies (1:5000 dilution). Membranes were washed in Tris-buffered saline (TBS), and signals were detected by enhanced chemiluminescence (ECL) according to the manufacturers’ instructions. *GAPDH* (Cell Signaling) was the internal control.

### TUNEL assay

Samples were fixed in 10 w/v PBS-buffered formaldehyde and embedded in paraffin. Sections (5 μm) were serially cut from each block. TUNEL staining was performed using a TUNEL detection kit according to the manufacturer’s instructions (Roche Diagnostics, Mannheim, Germany). Briefly, sections were rehydrated, incubated in 3 v/v H2O2 for 10 minutes, and then incubated with proteinase K (Roche) at 37 °C for 15 min. The TUNEL reaction mixture was added to the samples, and incubated at 37 °C for 60 min. Afterwards, converter-POD was added and samples were incubated at 37 °C for 30 minutes before being treated with diaminobenzidine tetrahydrochloride for 5 min. A negative control was treated in a similar manner except the TUNEL reaction mixture was omitted. For the quantitative analysis, the percentage of TUNEL-positive endometrial cells was recorded in each group, and analysis was repeated on sections from the same biopsy on three separate occasions.

### Immunohistochemistry

Briefly, 5-μm sections of the endometrium were lightly fixed with 4% paraformaldehyde in phosphate-buffered saline (PBS) for 10 min, washed in TBS, incubated with peroxidase (Biocare Medical, Concord, CA, USA) for 10 min to inhibit endogenous peroxidase activity, re-washed, and incubated with blocking serum for 20 min. The sections were treated as follows. The following primary and secondary antibodies were used: human NCAM-1/CD56 affinity-purified polyclonal antibodies (AF2408, R&D), mouse anti-human GnRHR monoclonal antibodies (ab24095, Abcam), human anti-FasL/TNFSF6 affinity-purified polyclonal antibodies (AF126, R&D), and rabbit anti-perforin polyclonal IgG (SC9105, Santa Cruz). Anti-rabbit and mouse horseradish peroxidase-conjugated secondary antibodies (Envision, Dako, K500711 kit) were used for detection. The primary antibodies were replaced with either PBS with 1.0% BSA or rabbit non-immune IgG as the negative control. Haematoxylin (blue) was used as a counterstain. The slides were visualized using a Nikon Eclipse 80i camera for bright-field imaging (Nikon Inc., Melville, NY, USA), and analysed with Image Pro Plus 6.0.

### Statistical analysis

The results were expressed as mean values ± standard deviation (SD) and compared with one-way analysis of variance (ANOVA) using SPSS 22.0 (IBM, Inc.). Data were considered statistically significance at *P* < 0.05.

## Additional Information

**How to cite this article**: Xu, B. *et al*. Increased Uterine NK cell numbers and perforin expression during the implantation phase in IVF Cycles with GnRH Antagonist Protocol. *Sci. Rep.* **7**, 39912; doi: 10.1038/srep39912 (2017).

**Publisher's note:** Springer Nature remains neutral with regard to jurisdictional claims in published maps and institutional affiliations.

## Supplementary Material

Supplementary Information

## Figures and Tables

**Table 1:** Demographic characteristics of women in the four groups.

	Natural group (n = 20)	Long agonist protocol group (n = 20)	Flexible antagonist protocol group (n = 20)	Fixed antagonist protocol group (n = 20)
Age (years)	29.7 ± 3.1	29.2 ± 2.6	33.2 ± 6.9	33.2 ± 6.9
Weight (kg)	61.1 ± 4.5	58.4 ± 6.7	59.3 ± 5.7	63 ± 5.2
Body mass index	22.9 ± 1.4	22.5 ± 1.9	20.6 ± 4.5	21.3 ± 3.5
Stimulation duration (day)	/	12.8 ± 6.6	10.4 ± 1.8	10.8 ± 0.6
Total Gn used (IU)	/	2167.5 ± 405	2047 ± 780	2377.5 ± 495
Average serum oestradiol values on human chorionic gonadotropin (hCG) day (pmol/L)	/	6215.4 ± 2613.6	5955 ± 3126.6	6127 ± 3629.6
Total Cetrotide used (mg)	/	/	0.5 ± 0.2^a^	1.3 ± 0.3
Duration of Cetrotide used (day)		/	3.7 ± 1.4^a^	5.3 ± 1.2
Average serum progesterone values on hCG day (ng/L)	/	1.4 ± 0.9	1.8 ± 1.2	2.3 ± 1.3
Average serum LH values on hCG day (U/L)	/	1.0 ± 0.7^b^	2.1 ± 1.7	2.2 ± 1.2
Number of oocytes retrieved	/	13.8 ± 6.2	14.3 ± 6.6	14.3 ± 7.5
Endometrial thickness on biopsy day (cm)	1.0 ± 0.1	0.9 ± 0.2	0.9 ± 0.1	0.8 ± 0.1

^a^P < 0.01 compared with the fixed GnRH antagonist group; ^b^P < 0.05, compared with the fixed and flexible GnRH antagonist groups.

**Table 2:** Microarray analysis of endometria during the window phase of three groups.

Patient ID	Age (year)	Infertility Duration (year)	Cause of infertility	IVF/ICSI	BMI	Study group	Time of biopsy
1	25	2	Fallopian tube blockages	IVF	18.9	untreated	LH + 7
2	31	5	Oligospermia	ICSI	22.5	untreated	LH + 7
3	32	3	Oligospermia	ICSI	20.8	untreated	LH + 7
4	29	2	Oligospermia	ICSI	21.1	GnRH antagonist	7 days after opu
5	27	4	Fallopian tube blockages	IVF	23.0	GnRH antagonist	7 days after opu
6	35	5	Fallopian tube blockages	IVF	19.7	GnRH antagonist	7 days after opu
7	24	1	Oligospermia	ICSI	24.2	GnRH agonist	7 days after opu
8	31	5	Fallopian tube blockages	IVF	22.0	GnRH agonist	7 days after opu
9	27	3	Fallopian tube blockages	IVF	24.2	GnRH agonist	7 days after opu

The control subjects had natural cycles with no treatment, and endometrial biopsies were obtained on day 7 after ovulation. For the GnRH antagonist and long agonist groups, endometrial biopsies were obtained on day 7 after oocyte recovery.

**Table 3:** Primers used in quantitative reverse transcriptase-polymerase chain reaction.

Primer	Sequence (from 5′ to 3′)
FASLGF	CTGGGGGCAGTGTTCAATCT
FASLGR	AAGACAGTCCCCCTTGAGGT
ITGALF	ACTGTAAGAGGCCAAAGGGC
ITGALR	CTGGTCACACGTTCGAGACA
ITGB2F	TGCGTCCTCTCTCAGGAGTG
ITGB2R	GGTCCATGATGTCGTCAGCC
KLRK1F	CAAGATCTTCCCTCTCTGAGC
KLRK1R	CATCTCCCAGCTGTGTCGAG
LCKF	GGAGGACCATGTGAATGGGG
LCKR	CATTTCGGATGAGCAGCGTG
MAP2K2F	CCCTGCCTCTCGGACTC
MAP2K2R	ACCGAGTAATGTGTGCCCTG
PRF1F	AGTGATGTGAGTGGTGGCTG
PRF1R	CATGGAGCTGGAATCCCGTA
TNFSF10F	TGAGAACCTCTGAGGAAACCA
TNFSF10R	TGCTCAGGAATGAATGCCCA
