# Supplementary material for: Increased Uterine NK cell numbers and perforin expression during the implantation phase in IVF Cycles with GnRH Antagonist Protocol
Source: Sci Rep. 2017 Jan 3;7:39912. doi: 10.1038/srep39912 (PMC5206673; doi:10.1038/srep39912)

Supplementary Information

Increased Uterine NK cell numbers and perforin expression during the implantation phase in IVF Cycles with GnRH Antagonist Protocol

Bufang Xu, Ph.D. [1], Jingwen Wang, M.S.[1], Lan Xia, M.S.[1], Dan Zhang, Ph.D.[1], Xian Wu, M.S.[1], Aijun Zhang, Ph.D. [1, 2]

1. Reproductive Medical Center of Ruijin Hospital, School of Medicine, Shanghai Jiao Tong University
2. Shanghai Key Laboratory of Reproductive Medicine

Correspondence: Dr Aijun Zhang (E-mail: zhaj1268@163.com)
Bufang Xu and Jingwen Wang contributed equally to this work.

Supplementary Fig. S1:

The full length SDS-PAGE gels and blots of total protein extracts from endometrial tissue with mAb FasL (A)、Perforin (B)、CD56 (C)、GnRHR (D).

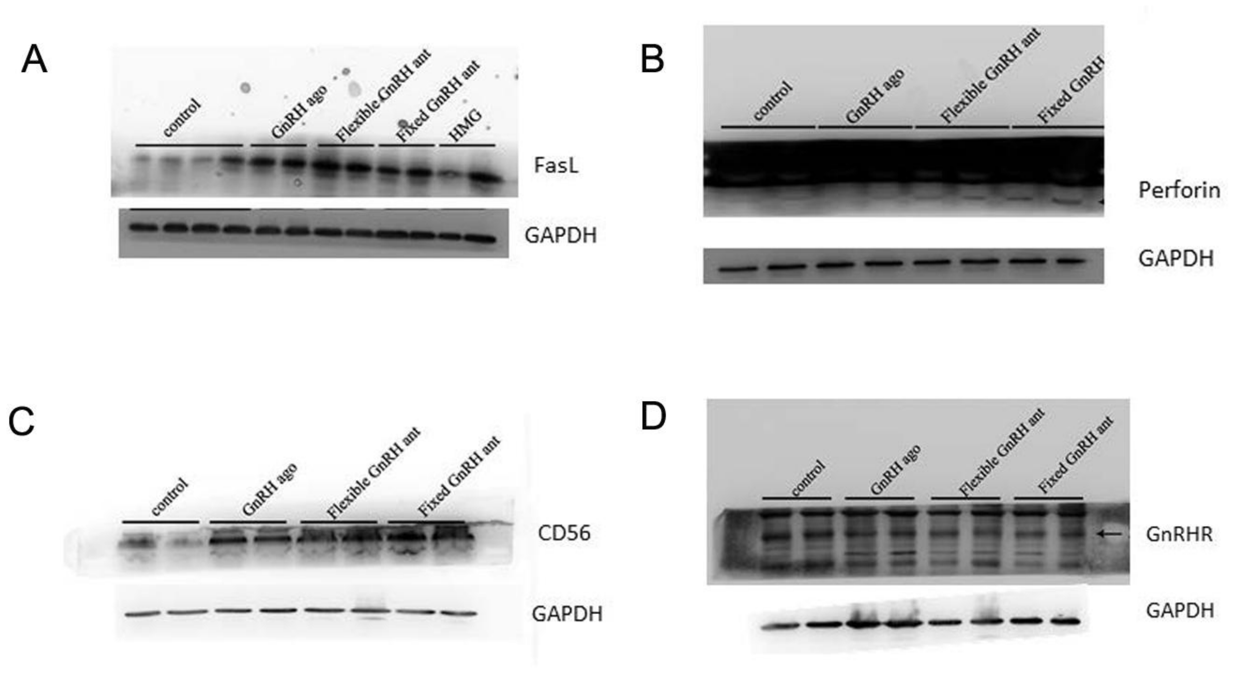

Supplement: Supplementary Information [file srep39912-s1.pdf]
